# Case Report: A rare case of post-traumatic intra-abdominal testicular migration mimicking torsion in a 9-year-old boy

**DOI:** 10.3389/fsurg.2026.1748517

**Published:** 2026-09-18

**Authors:** Ghadah Alsaleh, Wesam Khan, Khaled Bajaeifer, Mahmoud Saleh, Mohammed Alsakkaf, Shadi Alekish, Fatemah Alsaleh

**Affiliations:** 1 Global Center for Mass Gathering Medicine, Ministry of Health, Riyadh, Saudi Arabia; 2 Emergency Department, NEOM Hospital, Tabuk, Saudi Arabia; 3 Department of Surgery, Faculty of Medicine, University of Tabuk, Tabuk, Saudi Arabia; 4 Urology Department, Neom Hospital, Tabuk, Saudi Arabia; 5 General Surgery Department, NEOM Hospital, Tabuk, Saudi Arabia; 6 Medical Intern, College of Medicine, King Saud University, Riyadh, Saudi Arabia

**Keywords:** intra-abdominal migration, orchidopexy, pediatric, scrotal trauma, traumatic testicular dislocation, urology

## Abstract

**Background and objectives:**

Traumatic intra-abdominal testicular migration is an exceptionally rare urological emergency in pediatric patients, with limited literature to guide management. We present a case of a 9-year-old boy with post-traumatic intra-abdominal testicular displacement mimicking torsion, highlighting the diagnostic challenges and multidisciplinary approach required for optimal outcomes.

**Case presentation:**

A previously healthy 9-year-old male presented with severe right scrotal pain and empty hemiscrotum following blunt trauma during football. Initial ultrasound revealed a non-scrotal right testis with diminished vascularity, while CT confirmed intraperitoneal localization near the right inferior epigastric artery. Emergency laparoscopic exploration identified a viable intra-abdominal testis, managed successfully with combined laparoscopic mobilization, herniotomy, and open orchidopexy. The patient recovered uneventfully with normal testicular position at discharge.

**Conclusion:**

This case demonstrates that traumatic intra-abdominal testicular migration requires high clinical suspicion, timely imaging, and urgent surgical intervention**.** Despite the absence of pre-existing cryptorchidism, violent trauma can cause complete testicular displacement, emphasizing the need for thorough evaluation of pediatric scrotal trauma.

## Introduction

Traumatic testicular dislocation is a rare urological emergency. The condition typically results from high-impact blunt trauma to the scrotum, often occurring during motor vehicle accidents, sports injuries, or direct blows to the groin (1, 2). The sudden force can propel the testis from its normal scrotal position into the inguinal canal or, in extremely rare instances, into the abdominal cavity (3–5). This phenomenon is distinct from congenital cryptorchidism, as it involves previously descended testes that are traumatically displaced (6).

The pathophysiology of traumatic testicular dislocation is not fully understood but is thought to involve disruption of the gubernaculum and spermatic cord structures, allowing the testis to migrate from its anatomical bed (7, 8). While the inguinal canal is the most common site of dislocation, intra-abdominal migration is a rare variant. Potential mechanisms include a sudden traumatic increase in intra-abdominal pressure forcing the testis through a patent processus vaginalis or a weakness in the inguinal musculature (2, 9). Furthermore, predisposing anatomical factors may play a role, such as a patent processus vaginalis, inherent weakness of the inguinal musculature, a history of retractile testis, or a specific trauma kinetic vector, though these hypotheses require further investigation.

The clinical diagnosis remains challenging due to the condition's rarity and nonspecific presentation, which can lead to dangerous delays (10). Prompt recognition and intervention are paramount, as complications such as testicular ischemia, atrophy, or necrosis can develop rapidly without timely treatment (11, 12). Although ultrasound is the first-line imaging modality, its limited sensitivity for intra-abdominal testes means that clinical suspicion must guide the need for advanced cross-sectional imaging (CT or MRI) to achieve definitive localization and expedite surgical planning (13). The cornerstone of management is urgent surgical exploration, which allows for reliable testicular relocation, assessment of viability, and management of associated injuries, thereby minimizing long-term complications (14, 15).

Several factors contribute to the rarity of traumatic testicular dislocation into the abdominal cavity: the protected anatomical position of the testes, the strength of supporting structures in healthy individuals, and the specific vector of force required to propel the testis (2, 3). Most documented cases occur in adults following high-velocity trauma, making pediatric presentations exceptionally unusual (14, 16–18). The scarcity of reported cases in pediatrics has resulted in limited evidence to guide management, with each new instance providing valuable insights into this poorly understood phenomenon.

## Case presentation

### Patient history & clinical findings

A 9-year-old male with no significant past medical history and no prior history of undescended testis, presented to the Emergency Department (ED) with severe right-sided scrotal pain that had been progressively worsening over the past 7 h. According to the patient and his mother, the pain began after a blunt trauma to his groin during a football game when a friend accidentally punched him in the testicular area. Initially, the pain was mild, but it intensified significantly over the next few hours, radiating to the right lower abdomen and pelvic region. The mother reported that prior to the injury, both testes were clearly visible and palpable during bathing, but since the trauma, the right testis could no longer be felt or seen, raising concerns about possible retraction or ascent.

Upon arrival at the ED, the child was in obvious distress, crying due to the pain. Examination revealed mild redness of the scrotum, but the right hemiscrotum was completely empty, the right testis was non-palpable even when the patient was standing or performing a Valsalva maneuver (Figure 1). There was no significant swelling or bruising (ecchymosis) noted. The left testis was normally positioned and non-tender. Abdominal examination revealed mild tenderness in the right lower quadrant, but no signs of guarding or rebound tenderness. Clinical examination of the joints did not reveal any features of hypermobility. Due to the concerning findings, the patient was urgently referred for further evaluation and potential surgical intervention.

**Figure 1:**
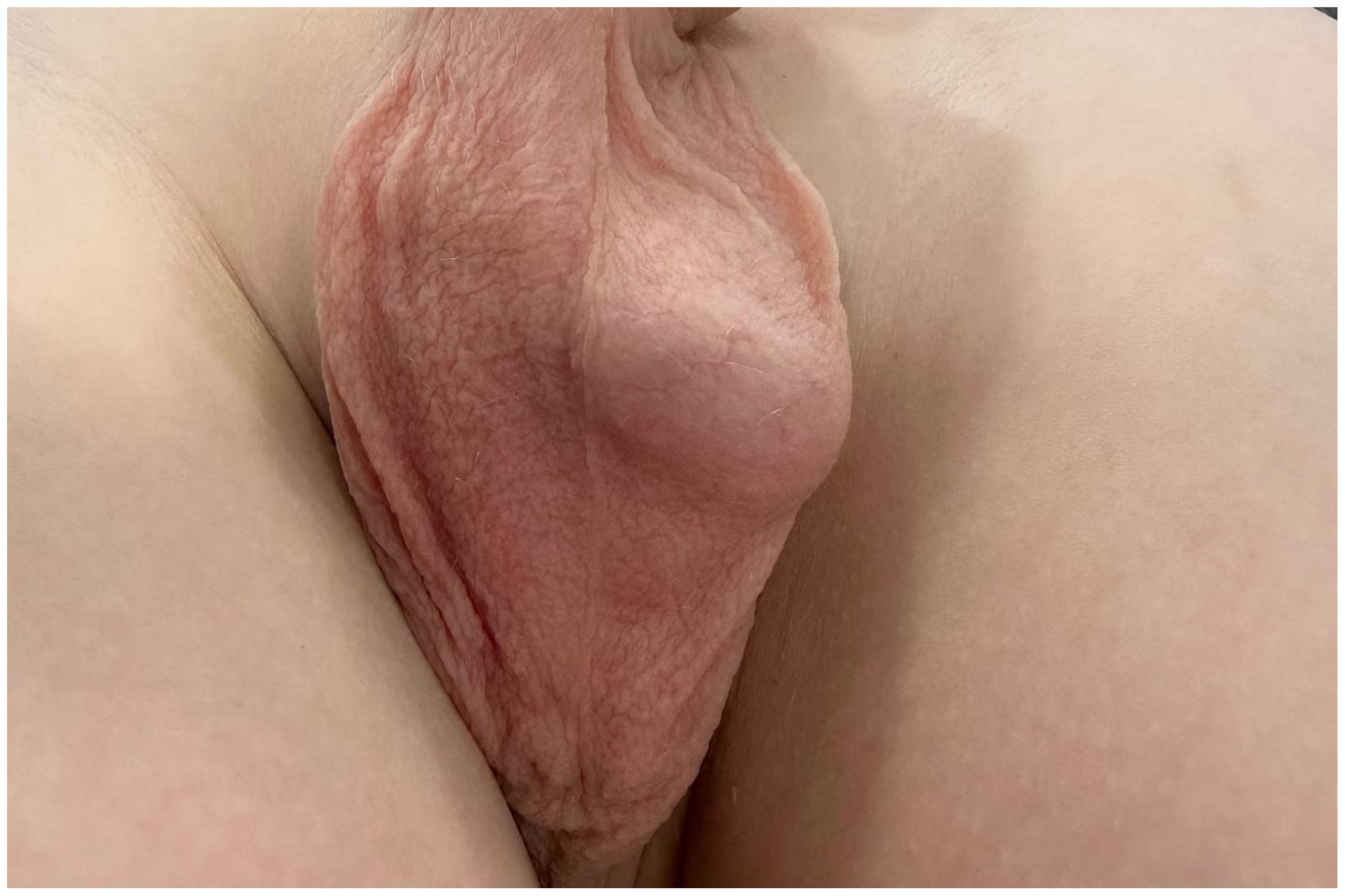
Clinical photograph of the empty right hemiscrotum upon presentation. The absence of the testis followed blunt perineal trauma, with subsequent surgical exploration confirming intra-abdominal migration.

### Diagnostic assessment & management

Given the high suspicion for testicular torsion or traumatic displacement, the patient underwent an urgent scrotal ultrasound, which revealed that the right testis was not located in the right hemiscrotum. Instead, a small (1.4 × 1.4 cm), slightly hypoechoic structure was identified in the right inguinal region, showing decreased vascularity, a finding highly suggestive of either an undescended testis or possible torsion (Figure 2). The left testis appeared normal in size (2.1 × 0.8 cm) with, homogenous echogenicity. No focal lesion, hydrocele, varicocele, or hernia was detected.

**Figure 2:**
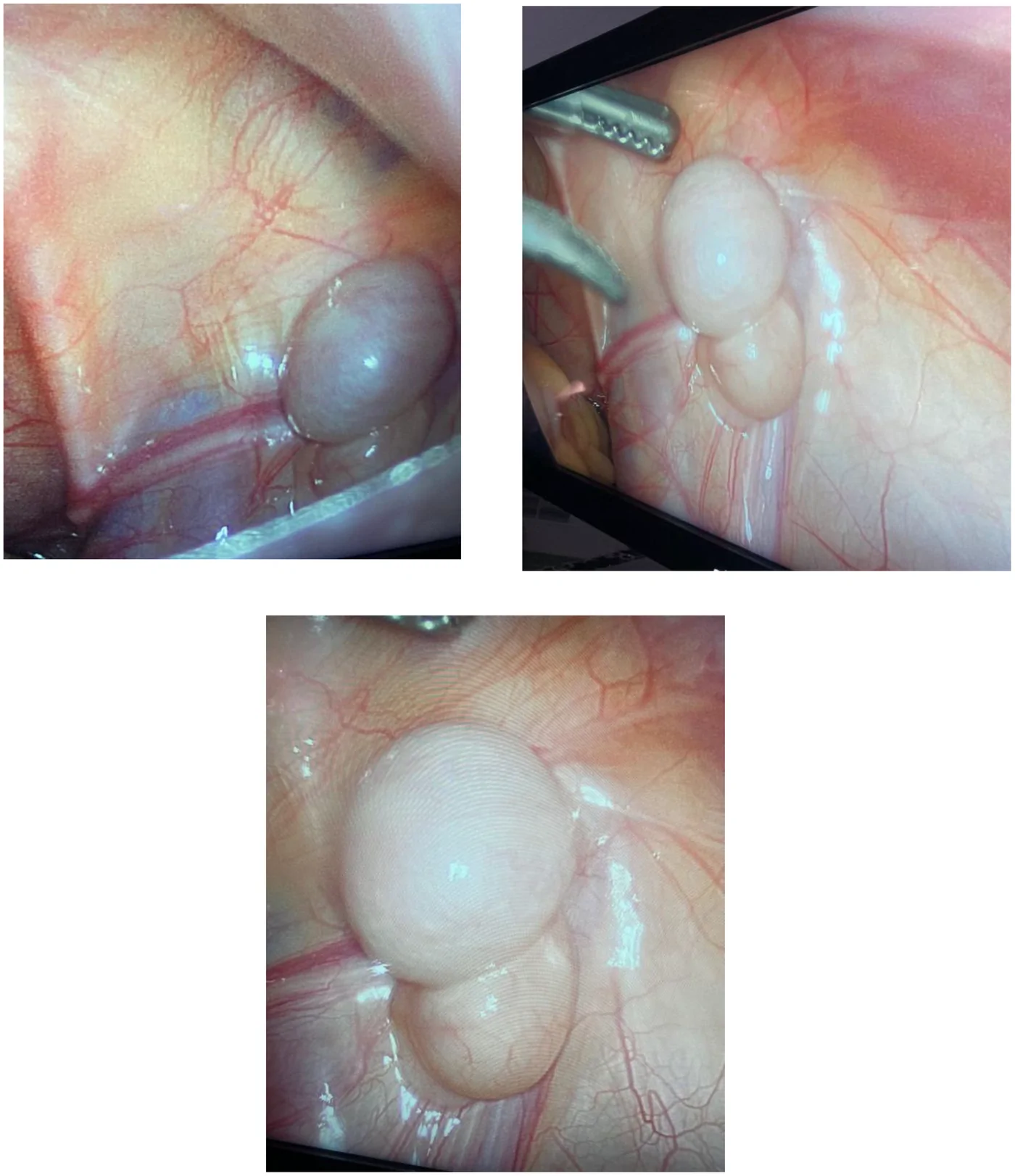
Incarcerated testes in inguinal canal.

A CT scan of the abdomen and pelvis with contrast was performed for better localization. The scan confirmed that the right testis was intraperitoneal, measuring 1.3 × 2 cm, positioned just above the origin of the right inferior epigastric artery. No other abdominal abnormalities were noted. The imaging findings confirmed traumatic intra-abdominal migration of the right testis. This stepwise imaging pathway was critical. The initial Doppler ultrasound confirmed the testis was absent from the scrotum and showed diminished vascularity, raising suspicion for displacement or torsion, but could not definitively localize the testis due to its deep position. The subsequent contrast-enhanced CT precisely localized the intraperitoneal testis, excluded associated injuries, and directly guided surgical planning. The hypoechoic inguinal structure seen on ultrasound likely represented the spermatic cord or a partially displaced component, explaining the apparent discrepancy with the definitive CT localization. Furthermore, the CT imaging provided clear anatomical visualization that was instrumental in counseling the family, demonstrating testicular viability and location, and facilitating informed consent for emergency surgery.

The parents were informed about the risks of testicular loss due to possible torsion and consented to surgery.

During surgery, laparoscopy confirmed that the right testis was viable and located intra-abdominally near the bowel (Figure 3). Detailed intraoperative findings revealed: (1) an incidental patent processus vaginalis with an open internal inguinal ring; (2) the displaced testis demonstrated preserved vascularity, with normal color and brisk bleeding response upon incision, confirming viability; (3) the spermatic cord structures (vas deferens and testicular vessels) were intact and demonstrated adequate elasticity, allowing mobilization without tension; (4) the gubernaculum testis appeared stretched but intact, suggesting traction rather than avulsion; and (5) the testis remained mobile and could be repositioned into the scrotum without vascular compromise. The testis was carefully mobilized, and a herniotomy was performed to repair the incidental inguinal hernia, with closure of the deep inguinal ring using Monocryl 3–0 sutures (Figure 4). Orchidopexy created a subdartos pouch in the right hemiscrotum and secured the testis in place with absorbable sutures to prevent future retraction.

**Figure 3:**
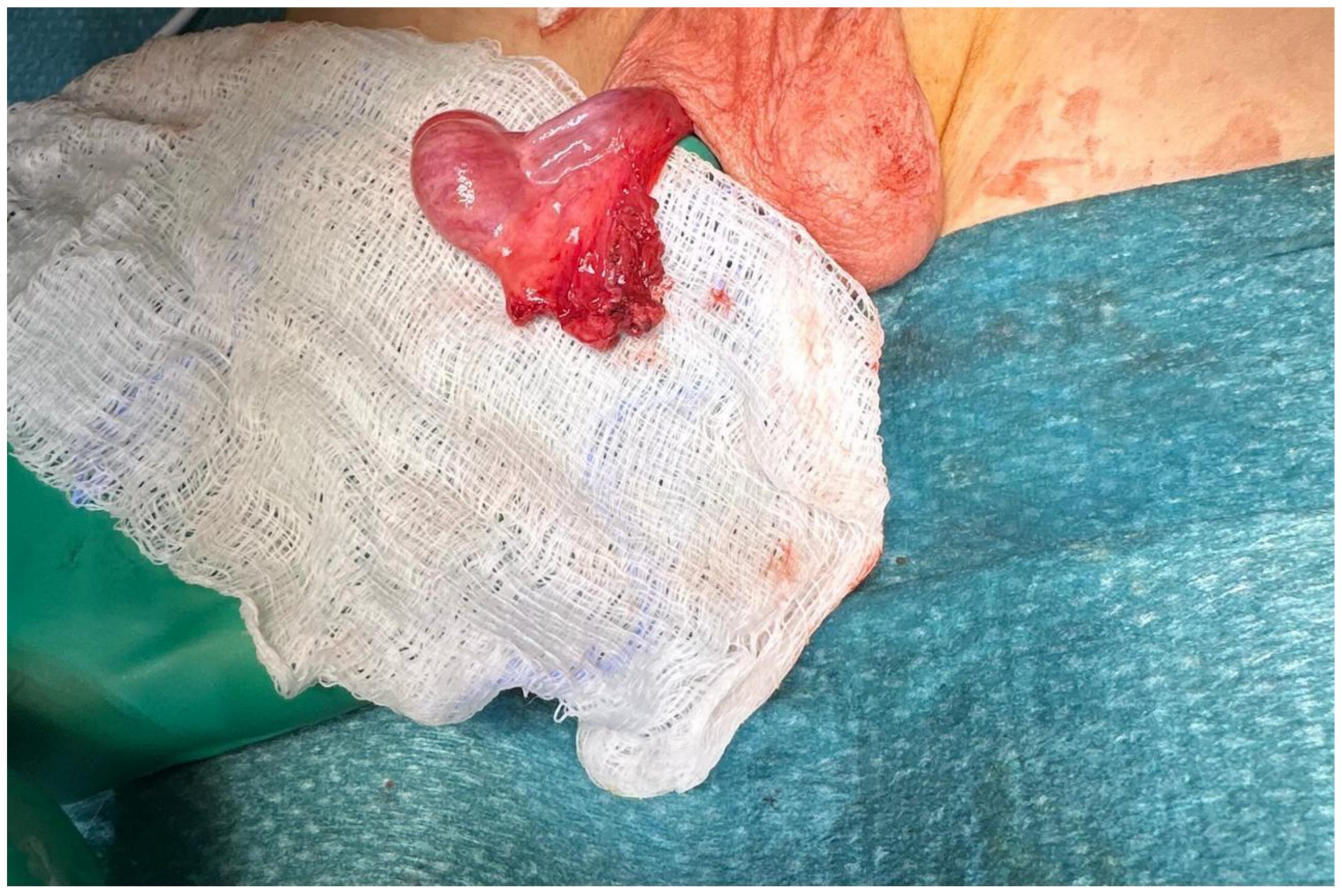
Intraoperative image obtained during right scrotal exploration demonstrating the testis retrieved from the intra-abdominal location. The testis appeared viable and was carefully mobilized, confirming post-traumatic intra-abdominal migration rather than torsion or agenesis.

**Figure 4:**
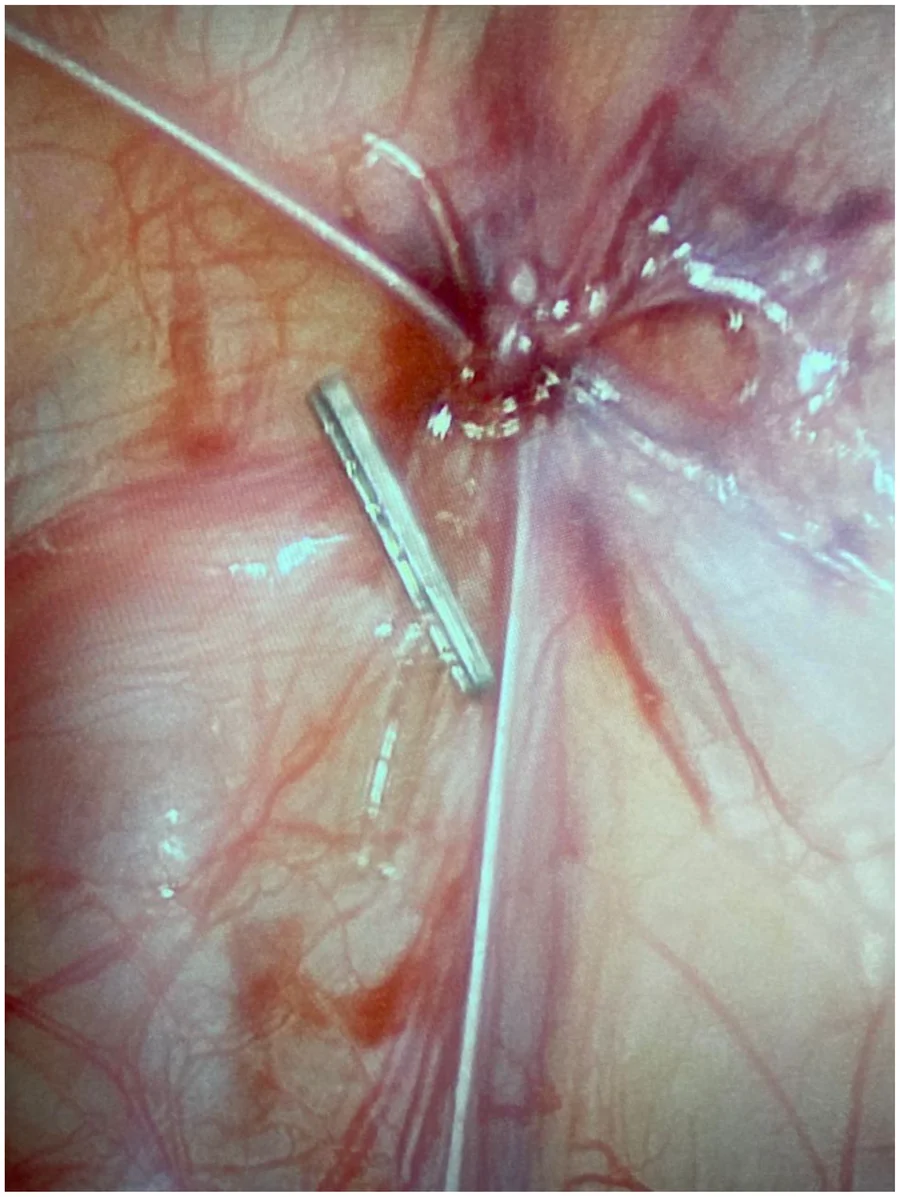
Laparoscopic herniotomy.

### Outcome & follow-up

By the first postoperative day, he was pain-free, tolerating a soft diet, and able to mobilize without difficulty. Both testes were now correctly positioned in the scrotum, and the patient was voiding normally (Figures 5, 6). The parents were counseled on postoperative care, including wound hygiene and the importance of follow-up.

**Figure 5:**
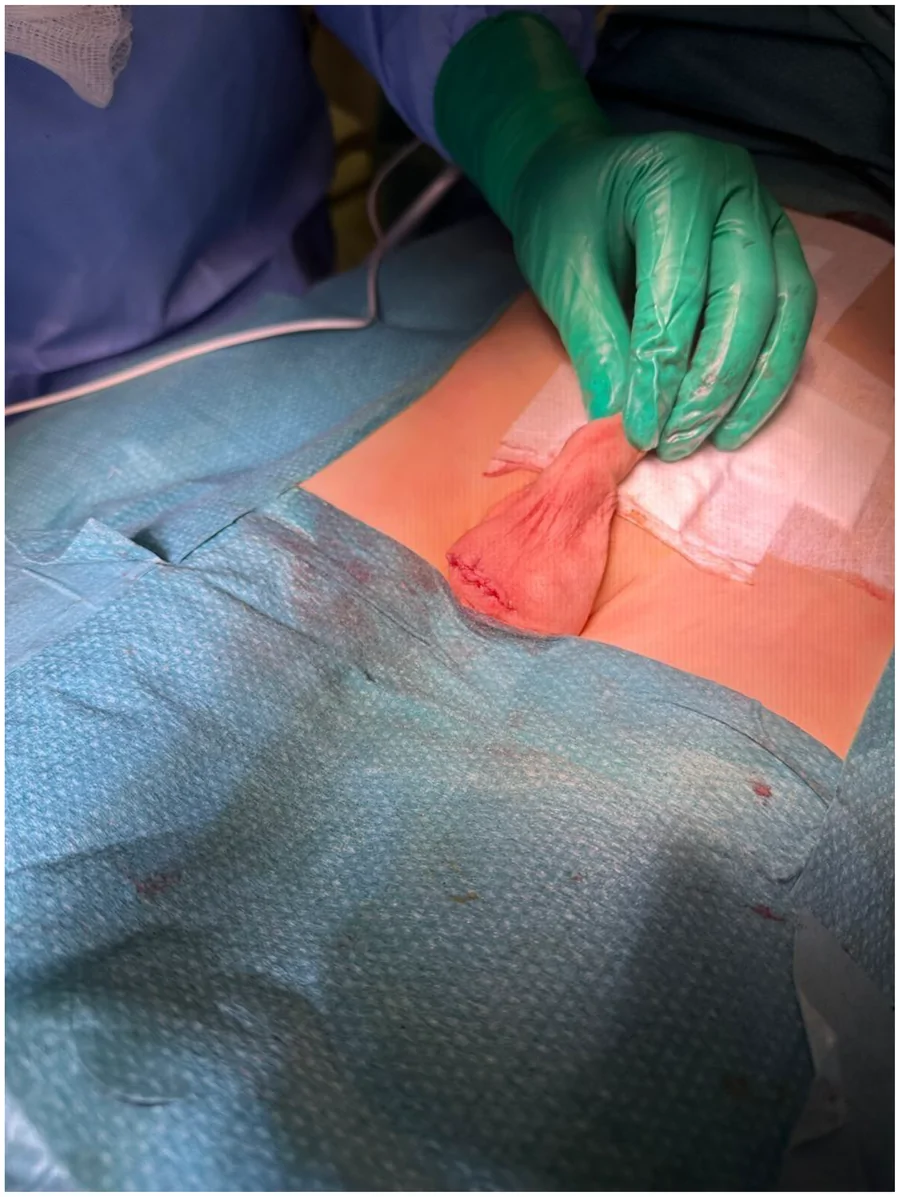
Post-orchidopexy image demonstrating the right testis successful repositioned into the scrotum with secure fixation. The wound closure was performed in layers, including dartos and skin, ensuring proper anatomical reconstruction and hemostasis.

**Figure 6:**
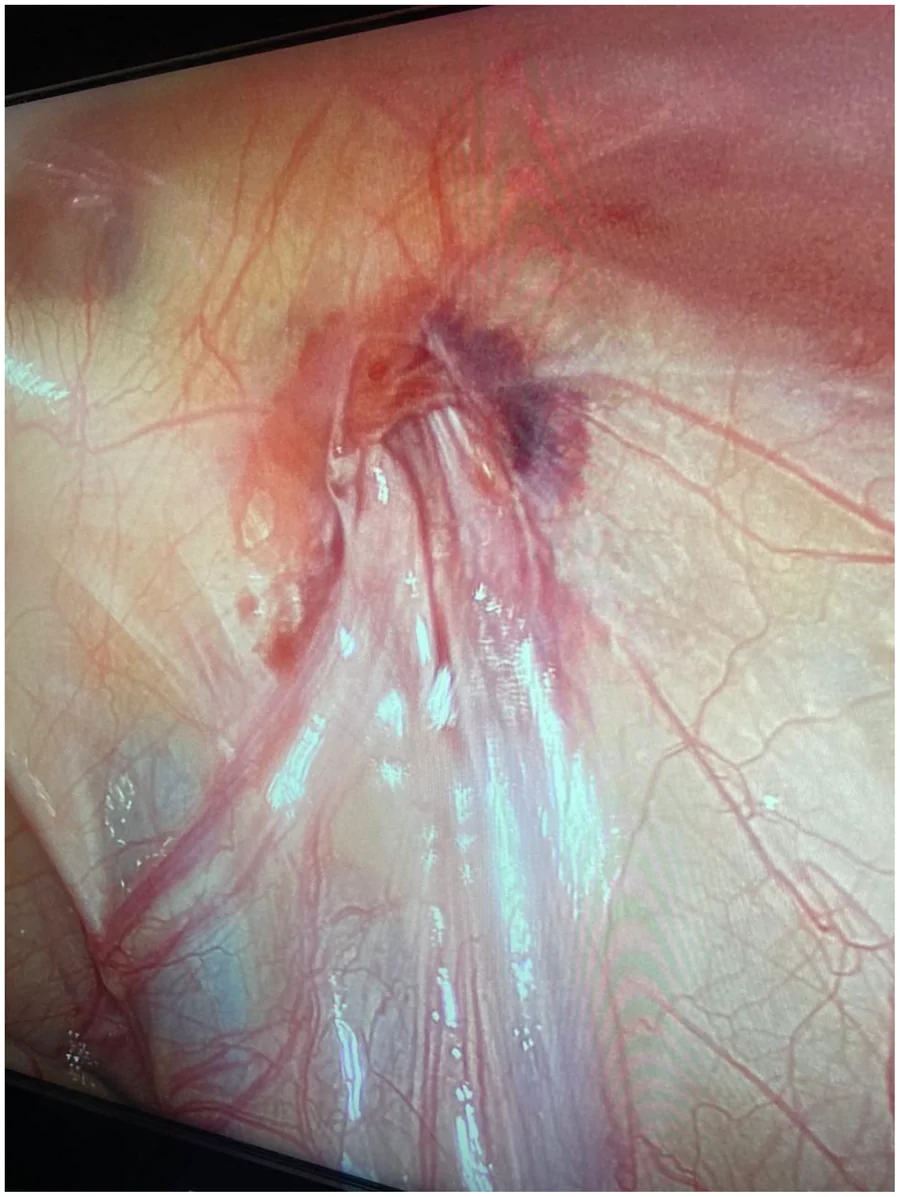
Inguinal area after tests relocation in the scrotum.

## Discussion

Traumatic intra-abdominal testicular migration poses significant diagnostic and therapeutic challenges, particularly in pediatric patients (3, 19). This report of a 9-year-old boy with post-traumatic displacement, initially mimicking torsion, presents several critical learning points that warrant careful consideration. The rarity of this presentation is defined by two key factors: the intraperitoneal location of the displaced testis, which is uncommon among traumatic dislocations, and the clinical scenario of acute torsion-like symptoms in a child with a confirmed history of previously descended testes and no cryptorchidism. This combination, along with its inherent diagnostic challenges, makes this report particularly valuable for clinicians.

Several clinical features made the diagnosis particularly challenging. While testicular torsion was the primary concern given the acute pain and absent testis on examination, several clinical features suggested an alternative pathology. The absence of significant scrotal swelling or ecchymosis argued against severe local trauma or hematoma formation. The progressive nature of the pain, radiating to the right lower quadrant, raised suspicion for testicular displacement rather than pure torsion. This clinical presentation aligns with Matzek & Linklater's (2013) pediatric case of a 10-year-old who suffered testicular dislocation after minor “monkey bar” trauma, though their patient showed inguinal rather than intra-abdominal migration (20). Notably, both cases lacked pre-existing cryptorchidism, challenging the assumption that testicular dislocation requires anatomical predisposition. This highlights the importance of maintaining a broad differential diagnosis when evaluating scrotal trauma, particularly when findings don't perfectly fit common conditions like torsion or hematoma.

The initial scrotal ultrasound, while failing to locate the testis in its normal position, provided the crucial clue of a hypoechoic structure in the inguinal region with diminished vascularity. The subsequent CT scan proved invaluable in precisely localizing the intra-abdominal position of the testis and confirming its relationship to surrounding structures. The imaging sequence in this case suggests that while ultrasound remains the first-line investigation for acute scrotal pathology, clinicians should have a low threshold for advanced cross-sectional imaging when the testis is not visualized in its expected location and clinical suspicion for displacement remains high. This imaging sequence contrasts with Chiu & Lin's (2022) approach in their adult polytrauma case, where immediate CT identified testicular dislocation alongside life-threatening injuries (18). Colalillo et al. (2023) reported a traumatic intra-abdominal testicular dislocation where a large scrotal hematoma prevented adequate physical examination, and diagnosis was delayed due to prioritization of life-threatening polytrauma (arterial bleeding, pelvic fractures) (5). Naik et al.'s (2021) bilateral dislocation case highlights ultrasound's role in confirming vascularity, though they supplemented with CT to rule out other injuries (21). In this acute trauma setting, CT was performed to guide surgical approach selection (laparoscopic vs. inguinal exploration), estimate the testis distance from the deep inguinal ring, and exclude associated intra-abdominal injuries. Diagnostic laparoscopy alone would have been sufficient to identify the testis and proceed with definitive management, and we do not advocate routine CT for all suspected testicular dislocations. However, in this specific case with an empty hemiscrotum following blunt trauma, the additional anatomical information facilitated preoperative planning and family counseling. Imaging should always be individualized based on the clinical scenario, and laparoscopy remains the gold standard for both diagnosis and treatment.

Several important surgical considerations emerged during treatment. The decision to proceed with laparoscopic exploration allowed for both diagnostic confirmation and therapeutic intervention through a minimally invasive approach. The intraoperative findings of the testis located intra-abdominally near the bowel, without evidence of torsion or vascular compromise, support the theory that the traumatic force caused a true displacement rather than simply triggering torsion in a predisposed testis. This mechanism differs from Colalillo et al.'s (2023) case where migration occurred with scrotal hematoma (5), and from Soaid et al.'s (2023) pediatric case where one testis auto-reduced during anesthesia while the other required orchidectomy for necrosis (22). Subramaniam et al. (2020) employed a novel two-point orchidopexy via single incision for bilateral dislocations (23), while Wang et al. (2021) successfully treated a 5-month delayed diagnosis with urethral reconstruction and orchiopexy (10).

Regarding patient outcomes, the postoperative course and short-term follow-up were reassuring, with the testis maintaining normal position and the patient experiencing no complications. However, several long-term considerations remain uncertain. Christodoulides et al. (2020) reported normal function at 2-month follow-up after manual reduction and orchidopexy (24). Naik et al. (2021) demonstrated preserved fertility markers at 3 months post-op (21), while Wang et al. (2021) confirmed testicular viability after delayed repair (10), suggesting outcomes may depend on initial injury severity.

The case raises intriguing questions about injury mechanisms. The precise anatomical pathway by which the testis moved from the scrotum to an intra-abdominal position remains speculative. The sudden increase in intra-abdominal pressure during impact may have forced the testis through a patent processus vaginalis. The absence of a clinically apparent hernia before or after the event makes this latter explanation less likely but not impossible. These mechanistic possibilities gain support from Subramaniam et al.'s (2020) direct observation of gubernacular detachment during surgery (23), while Soaid et al.'s (2023) case of spontaneous reduction under anesthesia suggests some dislocations may involve temporary rather than complete structural disruption (22).

An additional management consideration relates to the role of contralateral testicular fixation. In this case, contralateral orchiopexy was not performed at the time of surgery because the intraoperative findings indicated post-traumatic intra-abdominal migration rather than an underlying anatomical predisposition such as a bell-clapper deformity. The entire testis along with its coverings was displaced upward toward the abdomen, a mechanism distinct from that which typically warrants contralateral fixation. Nevertheless, the role of prophylactic contralateral fixation in such atypical presentations remains debatable. This option was discussed with the patient's parents during postoperative follow-up, including the potential benefits and risks, with the understanding that elective fixation could be considered at a later stage if clinically indicated.

While this single case cannot establish definitive practice guidelines, it adds to our understanding of this unusual condition and may help guide clinical decision-making when similar cases present. Future research directions might include establishing a registry of traumatic testicular migration cases to better characterize risk factors, optimal imaging protocols, and long-term outcomes. The WOFAPS survey (Shehata et al. 2022) highlights how such collaborative data could address current controversies in management approaches, particularly regarding hormone therapy and contralateral orchidopexy (25). Additionally, biomechanical studies could help elucidate the precise mechanisms by which testes become displaced following trauma, potentially leading to preventive strategies.

### Strengths and limitation

The primary strength of this report is the detailed documentation of a rare pediatric urological emergency, providing a clear clinical pathway from presentation to management. A key learning point is the demonstration of intraoperative flexibility, where laparoscopic exploration facilitated diagnosis before proceeding with definitive open repair. The main limitations are inherent to the case report format: the findings are from a single patient, limiting generalizability, and the short follow-up period precludes conclusions about long-term testicular viability or recurrence risk.

## Conclusion

Although rare, traumatic testicular displacement representing acquired cryptorchidism should be considered following scrotal or inguinal trauma. Clinical suspicion is essential when an acute scrotum presents with a non-palpable testis, a finding that differs from the typical presentation of intrascrotal torsion. Recognition of this presentation should prompt urgent surgical evaluation and management to preserve testicular viability and prevent long-term complications.

## Implications for clinical practice

This case provides several specific, actionable insights for managing pediatric scrotal trauma:

1. High Index of Suspicion: Traumatic testicular dislocation should be suspected in any child with acute scrotal pain, an empty hemiscrotum, and a history of blunt trauma, even with previously normal testicular descent.
2. Imaging Strategy: Initial scrotal ultrasound is mandatory. If the testis is not visualized in the scrotum or shows abnormal vascularity, immediate cross-sectional imaging (CT or MRI) is required to locate the displaced testis and rule out intra-abdominal migration.
3. Avoiding Diagnostic Pitfalls: Common errors include misattributing the empty hemiscrotum to congenital cryptorchidism (retraction) or focusing solely on torsion. Concomitant lower abdominal tenderness should raise suspicion for displacement.
4. Definitive Management: Urgent surgical exploration is the cornerstone of management. A laparoscopic approach offers diagnostic confirmation, assessment of viability, and a pathway for definitive repair (mobilization, herniotomy, and orchidopexy) in a single procedure.
5. Collaborative Care: Optimal outcomes rely on prompt, coordinated involvement of emergency medicine, radiology, urology, and pediatric surgery to streamline diagnosis and intervention, minimizing the risk of testicular loss.

## Data Availability

The original contributions presented in the study are included in the article/Supplementary Material, further inquiries can be directed to the corresponding author.
